# Proportional Multiaxial Fatigue Behavior and Life Prediction of Laser Powder Bed Fusion Ti-6Al-4V with Critical Plane-Based Building Direction Variations

**DOI:** 10.3390/ma18215056

**Published:** 2025-11-06

**Authors:** Tian-Hao Ma, Yu-Xin Wang, Wei Zhang, Jian-Ping Zhao, Chang-Yu Zhou

**Affiliations:** School of Mechanical and Power Engineering, Nanjing Tech University, Nanjing 211816, China

**Keywords:** multiaxial fatigue, laser powder bed fusion, Ti-6Al-4V, critical plane, fatigue life prediction

## Abstract

Laser powder bed fusion (L-PBF) is an additive manufacturing technique that enables the fabrication of complex geometries through a layer-by-layer approach, overcoming limitations of conventional manufacturing. In this study, multiaxial low-cycle fatigue (MLCF) tests were conducted on L-PBF Ti-6Al-4V (Ti64) specimens built in four different orientations, selected based on critical plane orientations identified from rolled titanium. Under proportional strain-controlled loading, the cyclic softening behavior, mean stress response, and fracture mechanisms of the material were systematically investigated. The results show that L-PBF Ti64 exhibits a three-stage softening characteristic (continuous softening, stable, and rapid softening). Fatigue cracks primarily initiate from inner-surface lack-of-fusion defects. Crack propagation shows cleavage and quasi-cleavage characteristics with tearing ridges, river patterns, and multi-directional striations. Proposed KBMP life prediction model, incorporating λ and building direction parameters, was developed. The KBMP-λ model demonstrates optimal accuracy, providing a reliable tool for the design of L-PBF titanium components subjected to complex multiaxial fatigue loading with relative errors within 20%.

## 1. Introduction

Additive manufacturing (AM) has emerged as an innovative high-end manufacturing technology in the 21st century. It overcomes the limitations of conventional manufacturing (CM) in producing components with complex geometries by building structures layer by layer. This bottom-up fabrication approach significantly improves material utilization compared to CNC machining, demonstrating irreplaceable advantages in high-end sectors such as aerospace and biomedical engineering [1,2].

Titanium and its alloys are widely used across a broad range of industrial applications due to their exceptional properties, including high specific strength, excellent work-hardening capability, good ductility, toughness, and outstanding corrosion resistance [3,4,5]. However, these advantageous properties are accompanied by significant challenges in processing. AM Ti-6Al-4V (Ti64), particularly produced via laser powder bed fusion (L-PBF), has been extensively studied in the literature for aerospace and biomedical applications, which is mainly due to the capability of this technique in fabricating complex geometries with acceptable dimensional accuracy [6,7,8,9,10]. Among AM processes, L-PBF employs the thinnest layer thickness during fabrication, resulting in a superior as-built surface finish compared to other AM techniques [11].

Fatigue represents one of the most prevalent failure modes in mechanical structures. With the increasing demand for high-speed, high-load, and lightweight equipment, structural safety margins have been reduced, leading to a higher incidence of fatigue-related failures. Consequently, fatigue fracture remains a critical challenge in engineering applications [12]. The layer-wise nature of AM introduces pronounced microstructural anisotropy due to the building direction, which significantly influences the fatigue behavior of AM materials [13]. Additionally, intrinsic defects were generated during the AM process such as lack of fusion (LOF), porosity, and residual stress, further deteriorating fatigue performance [14,15].

Several studies have investigated the effects of building direction and defects on the fatigue behavior of L-PBF Ti64. Nicoletto et al. [16] reported that specimens fabricated in vertical and horizontal orientations exhibited shorter fatigue lives, particularly in the vertical direction, due to poor surface finish. Hartunian [17] studied the fatigue properties and fracture mechanisms of L-PBF Ti64 specimens built in both horizontal and vertical orientations, finding that vertically built specimens exhibited more pronounced fatigue degradation due to LOF defects forming between adjacent layers. Similarly, Persenot [18] demonstrated that surface morphology strongly depends on building direction, with specimens fabricated in vertical, horizontal, and diagonal directions exhibiting distinctly different surface features. Vertically built samples tended to have directional surface micro-notches, resulting in shorter fatigue lives. Chastand et al. [19] tested the alloy fabricated by selective laser melting (SLM) and found that anisotropy is negligible after 6000 cycles, except when the loading is parallel to the beam. Agius et al. [20] compared SLM and wrought alloys and reported that the second showed significantly higher plastic work than the SLM.

Although interest in the multiaxial fatigue behavior of AM materials has grown in recent years, there remain substantial research gaps, particularly concerning the influence of building direction under multiaxial loading. Fatemi [8,21] investigated the effect of surface texture on the torsional and multiaxial fatigue behavior of L-PBF Ti64 in the vertical build direction, finding that the maximum principal stress criterion effectively predicted crack orientation in brittle fracture scenarios. Carrion [6] explored the effects of layer orientation and surface roughness on multiaxial fatigue performance, revealing that fatigue cracks initiate at surface defects and grow along planes of maximum tensile stress. Under torsional loading, the fatigue performance was primarily influenced by the orientation of the deposited layers. Vertically built specimens, in which surface micro-notches were not aligned with the critical fatigue plane, demonstrated enhanced fatigue resistance.

Most existing studies on the fatigue behavior of L-PBF Ti64 have focused on a limited number of fixed building directions. Given the successful application of critical plane theory in analyzing CM materials, investigating the multiaxial fatigue behavior of L-PBF Ti64 with building direction variations based on critical plane orientations holds great promise. To address this research gap, this paper systematically investigates the multiaxial low-cycle fatigue (MLCF) behavior of L-PBF Ti64 at four different building direction angles (0°, 12°, 16°, 27°), chosen based on critical plane orientations identified from prior studies on rolled titanium alloys [22,23]. Strain-controlled MLCF tests were performed to obtain cyclic stress–strain responses and to assess cyclic hardening/softening behavior and mean stress evolution. Post-failure analysis of macroscopic fatigue crack propagation was conducted using high-resolution digital camera and scanning electron microscopy (SEM) to investigate fracture surface characteristics and underlying damage mechanisms. Furthermore, proposed KBMP models with different parameters are developed and validated to offer a fatigue life predictive methodology for L-PBF Ti64 under complex multiaxial loading conditions.

## 2. Materials and Experimental Procedures

**Figure 1:**
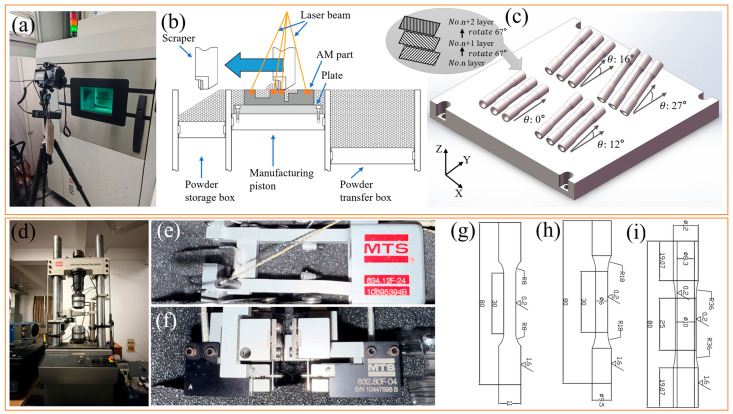
(**a**,**b**) EOS M290 L-PBF system, (**c**) building direction schematic, (**d**–**f**) MTS 809 material test system, and (**g**–**i**) test specimen dimensions.

Ti64 powder with a chemical composition conforming to ASTM F1472 [24] and ASTM F2924 [25] standards was utilized in this study. The Ti64 powder, supplied by EOS, had a nominal particle size distribution of approximately 63 μm. Specimens were fabricated in multiple batches using an EOS M290 L-PBF system (EOS GmbH, Krailling, Germany), with each batch oriented along a distinct building direction, as illustrated in Figure 1a–c. All L-PBF processes were conducted employing the standard parameters recommended by EOS, which are summarized in Table 1.

All tests were performed on an MTS 809 hydraulically driven material test system (MTS Systems Corporation, Eden Prairie, Minnesota, USA), depicted in Figure 1d–f. The tests were conducted at room temperature (20 °C), maintained by the laboratory’s air conditioning system. The system was controlled using the MTS Flex Test 40 Station Manager (Version 5.7E 5045). Strain was measured with an axial extensometer (model 634.12F-24) for uniaxial tests and an axial-torsional extensometer (model 632.80F-04) for multiaxial tests. All test data were acquired at a sampling rate of 300 Hz through the system’s control computer.

**Figure 2:**
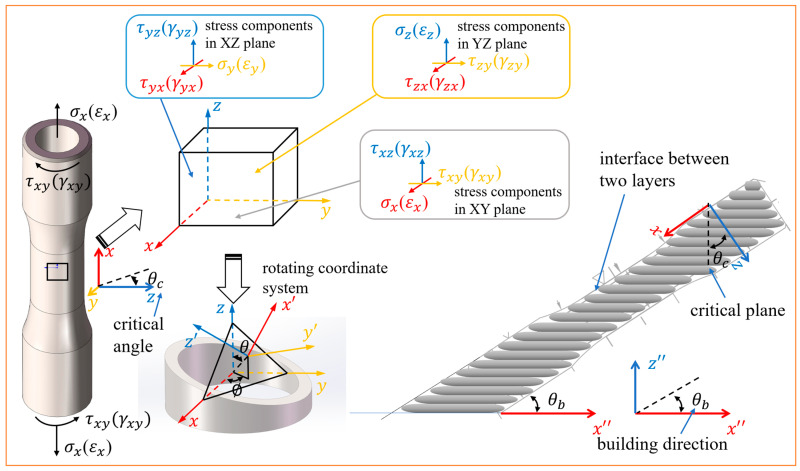
Schematic of critical plane and building direction.

The four building directions were selected based on our previous work [26], which applied critical plane theory to the MLCF behavior of a CM (rolled) titanium alloy. In recent years, the critical plane approach has gained widespread application in multiaxial fatigue research due to its strong alignment with experimental observations [22,23,27,28]. Under multiaxial loading conditions, fatigue cracks typically initiate and propagate along a specific angular plane, commonly referred to as the critical plane. In strain-controlled loading modes, the critical plane is typically defined as the plane of either maximum principal strain or maximum shear strain. In this study, the selected building directions are oriented at approximately 90° to the respective critical planes, as shown in Figure 2.

The mean fracture surface inclination and critical plane based on the maximum principal strain are 12.04°/16.88° with multiaxial strain ratio (λ) of 0.865 and 17.92°/26.58° with λ of 1.73, respectively. λ is the ratio of the torsional strain to the axial strain, as shown in Equation (1):

(1) $$\lambda =\frac{\Delta \gamma /2}{\Delta \varepsilon /2}\;$$

where Δε/2 and Δγ/2 are the axial and torsional strain amplitudes, respectively.

Considering that the loading cases applied in this paper are almost based on λ of 0.865 and 1.73, the final selected building directions were 0°, 12°, 16° and 27°. The 0° direction was included as a baseline reference, commonly used in prior studies, to assess the effect of small-angle deviations aligned with critical planes on multiaxial fatigue behavior. After fabrication, the specimens were separated from the direct base via wire-cutting, with heat-treated (2 h at 800 °C in argon atmosphere) and then machined by lathe processing to remove 0.5 mm thick inner and outer surfaces, respectively. The purpose of heat-treated was for stress relief annealing. The final specimen dimensions shown in Figure 1i represent the net dimensions after machining. The 3D models used in the L-PBF process were designed with positive dimensional allowances to compensate for the expected material removal during finishing.

Prior to the MLCF test, standard tensile and uniaxial low cycle fatigue (LCF) tests were conducted on specimens with different building directions to obtain the material’s static and dynamic mechanical properties. These tests were performed using the MTS 809 system under strain-controlled mode with axial extensometer. The specimen geometric dimensions for tensile and LCF tests are shown in Figure 1g,h. The surface roughness of the gauge length for tensile, LCF, and MLCF specimens was Ra 0.2, while the surface roughness of the clamping section was Ra 1.6. Tensile tests were conducted at a strain rate of 0.0005 s^−1^ with two replicate tests conducted per building direction. LCF tests were performed at axial strain amplitudes of 0.4%, 0.6% and 0.8% for each building direction. All tensile and LCF test results are summarized in Section 3.1 (Table 2 and Table 3).

MLCF tests were conducted under proportional, fully reversed loading (R = −1 for both axial and shear strains) using the MTS 809 system with an axial-torsional extensometer. R is the ratio of the peak strain amplitude to the valley strain amplitude. The frequency of MLCF tests was 1 Hz and phase angle was 0°. The applied axial strain amplitudes were 0.4% and 0.6% combined with λ of 0.865, 1.73, and 3.46 [26]. All strain amplitudes were directly controlled by the MTS MPE test suit via the dual-channel axial-torsional extensometer using a triangular waveform. Three additional specimens with different building directions were tested to assess fatigue life dispersion. MLCF test results are provided in Section 3.1 (Table 4).

## 3. Mechanical Behavior of L-PBF Ti64 with Different Building Directions

Mechanical behavior including static/dynamic mechanical properties, cyclic softening characteristics and mean axial and torsional stress response of L-PBF Ti64 with different building directions will be discussed in this section.

### 3.1. Static and Dynamic Mechanical Properties

A summary of the tensile, LCF, and MLCF tests and corresponding results is provided in Table 2, Table 3 and Table 4.

The tensile properties of L-PBF Ti64 across the four building directions are summarized in Table 2. Overall, the tensile strength and yield strength of L-PBF Ti64 are slightly higher than those of EMB Ti64, while the elastic modulus is slightly lower than that of EMB Ti64 [29,30]. The mean ratio for $\sigma_{u}/\sigma_{y}$ of L-PBF Ti64 is 1.16, suggesting a tendency for cyclic softening [29]. The effect of building direction on uniaxial fatigue life is presented in Table 3. To obtain the dynamic mechanical properties of L-PBF Ti64, the Ramberg–Osgood model [31] was developed to describe a material’s dynamic mechanical behavior for the elastic and plastic regions of the stress–strain curve, as shown in Equation (2):

(2) $$\frac{\Delta \varepsilon }{2}=\frac{\Delta \sigma }{2E}+{\left(\frac{\Delta \sigma }{2K^{\prime }}\right)}^{\frac{1}{n^{\prime }}}\;$$

Established empirical rules for the Ramberg–Osgood parameters suggest that a material is prone to cyclic softening when $\sigma_{u}/\sigma_{y}$ < 1.2 and $n^{\prime }$ < 0.2 [32]. Furthermore, the value of E$K^{\prime }$/(E + $K^{\prime }$) should approximate $K^{\prime }$. The fitted parameters for Equation (2) with different building directions are as follows: $\;n^{\prime }=\;0$.188, $K^{\prime }$ = 1003.91 in 0°, $n^{\prime }$ = 0.176, $K^{\prime }$ = 926.50 in 12° and $n^{\prime }$ = 0.181, $K^{\prime }$ = 963.84 in 27°. As a comparison, Benz et al. [33] reported 0.012 and 1022, respectively, for EBM Ti64.

Table 4 details the applied axial and torsional strain amplitudes, half-life response stress amplitudes, and fatigue life distributions for each specimen in the MLCF test. The repeatability of the fatigue life (Nf) was validated using three sets of duplicate specimens. The observed Nf values for these pairs were as follows: AMPMF02 vs. AMPMF02-R: 4752 vs. 5003, AMPMF06 vs. AMPMF06-R: 8982 vs. 8164 and AMPMF10 vs. AMPMF10-R: 1394 vs. 1176. The deviation in fatigue life under identical loading conditions remains within 10%, confirming good experimental consistency.

### 3.2. Cyclic Softening/Hardening Characteristics

**Figure 3:**
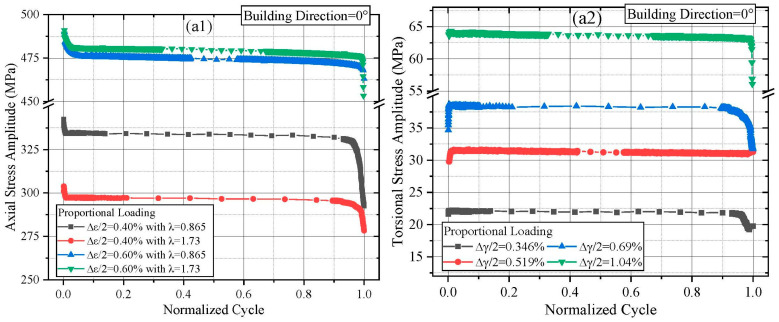

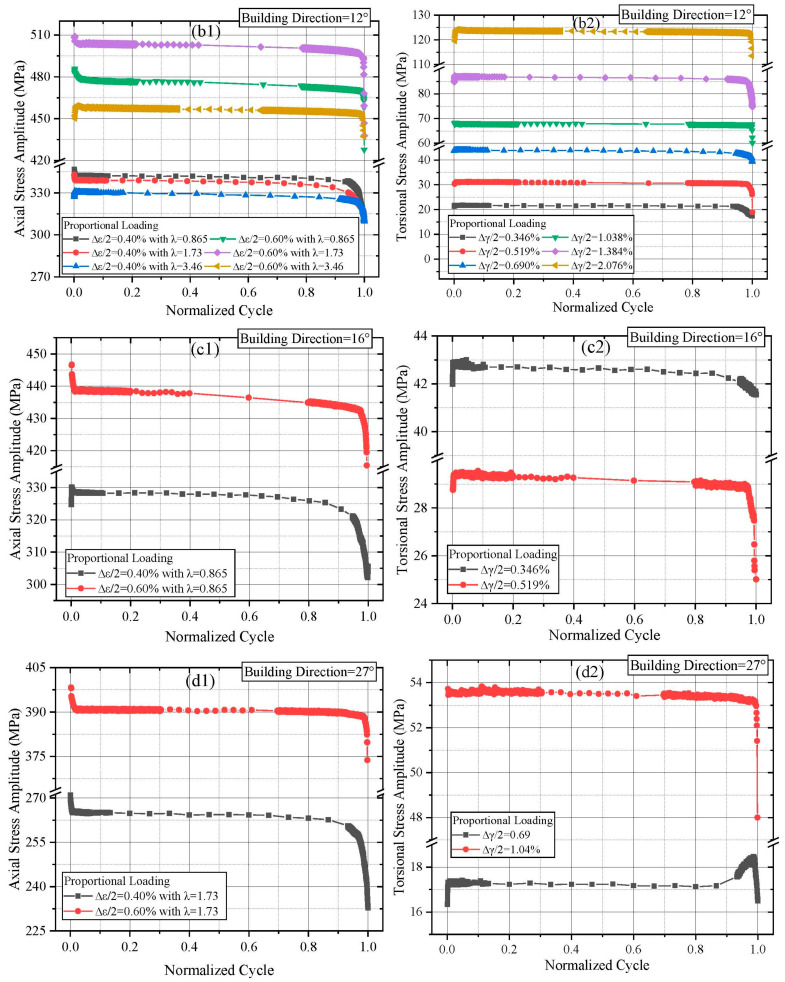
Axial and torsional stress amplitude response of L-PBF Ti64 with different building direction: (**a1**,**a2**) building direction = 0°, (**b1**,**b2**) building direction = 12°, (**c1**,**c2**) building direction = 16°, (**d1**,**d2**) building direction = 27°.

The cyclic softening/hardening behavior of materials under MLCF loading can be characterized by tracking the evolution of axial and torsional stress amplitudes with the number of cycles. Figure 3 presents these variations for L-PBF Ti64 specimens fabricated at different building directions (all figures were prepared using OriginPro 2018). To facilitate a clearer comparison of cyclic behavior across different loading conditions, the fatigue cycle life data are normalized.

**Figure 4:**
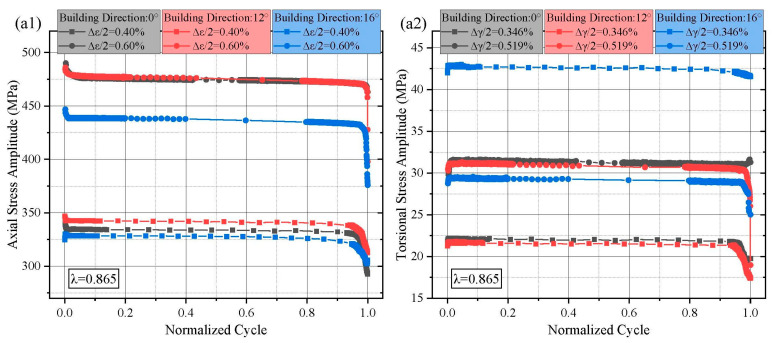

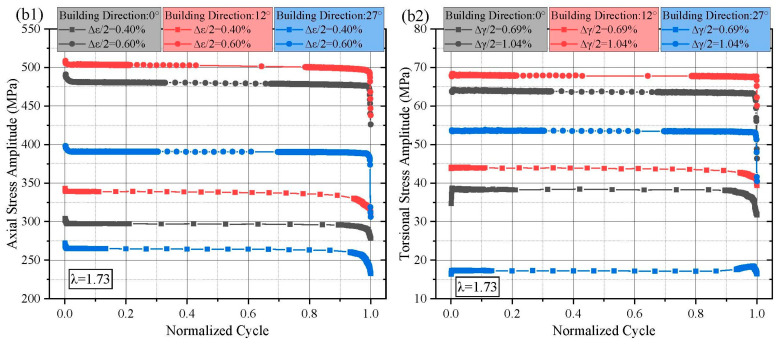
Axial and torsional stress amplitude response of L-PBF Ti64 with different λ: (**a1**,**a2**) λ = 0.865, (**b1**,**b2**) λ = 1.73.

As shown in Figure 3(a1), specimens built at 0° exhibit the classical three-stage (continuous softening, stable, and rapid softening) softening characteristic of titanium alloys. At this orientation, specimens subjected to higher multiaxial strain ratios exhibited more pronounced softening at the 0.4% axial strain level. However, for an axial strain loading level of 0.6%, varying the multiaxial strain ratios had a limited effect on the stress response. In Figure 3(b1), specimens built at 12° and tested under λ = 0.865 and 1.73 similarly exhibited three-stage softening. An initial transient hardening was observed when the strain ratio increased to λ = 3.46. Compared to the 0° orientation, the 12° orientation showed slower stress amplitude decay under 0.4% axial strain with increasing λ. For 0.6% axial strain, the stress response showed a brief hardening phase before transitioning to softening as λ increased. It is worth mentioning that the unexpected peaks can be observed prior to failure occurred in the torsional stress responses, as shown in Figure 3(d2) and Figure 4(b2). Unlike axial stress responses, where positive and negative values clearly represent tensile and compressive stresses, in torsional stress responses, positive and negative values merely indicate clockwise and counterclockwise directions. Ideally, the positive and negative values of torsional stress responses have the same effect on material failure. Additionally, since the torsional stress amplitude is calculated as (torsional peak stress − torsional valley stress)/2, under ideal conditions, the likelihood of a specimen’s crack propagation path developing in either the clockwise or counterclockwise direction before failure is equal. Furthermore, under multiaxial loading, crack surfaces are typically inclined rather than planar. When significant obstacles hinder crack growth, torsional stress in either direction may transiently surge, leading to a brief peak in the amplitude response just before failure.

Owing to limited availability of Ti64 powder, specimens built at 16° and 27° were primarily used for fatigue life analysis rather than in-depth stress evolution studies. Overall, most of these specimens also exhibited the three-stage softening pattern, with one specimen showing an additional initial hardening phase. Regarding the torsional stress response, most specimens showed an initial transient hardening instead of continuous softening, while the subsequent stable and rapid softening stages remained consistent with the axial response. The torsional stress response was generally monotonic with respect to the applied torsional strain amplitude, as expected. To more clearly illustrate the effects of building direction on stress response at fixed strain ratios, Figure 4 presents an alternate view of the stress amplitude distributions under λ = 0.865 and λ = 1.73.

At λ = 0.865 and 0.4% axial strain, the highest axial stress response at half-life (340 MPa) was observed in the 12° specimens, followed by the 0° (330 MPa) and 16° (325 MPa) specimens. At 0.6% axial strain, the axial stress responses of the 0° and 12° specimens were nearly identical and significantly higher than that of the 16° specimens (475 MPa vs. 435 MPa at half-life). In the torsional channel, the 16° specimens exhibited a uniquely high stress response (43 MPa at half-life) under 0.346% torsional strain. Other specimens demonstrated more consistent and comparable torsional stress responses. When λ increased to 1.73, both axial and torsional stress responses exhibited greater variability than those at λ = 0.865. Under this condition, the 12° specimens consistently showed the highest stress responses, followed by the 0° and 27° specimens in descending order.

For materials that exhibit cyclic softening, higher stress response is generally associated with longer fatigue lives, as more cycles are required before the final rapid softening stage initiates. Although the variations in building direction in this study were relatively small, the distinct differences in stress response under identical loading conditions indicate that even minor deviations in building direction, when aligned with critical planes, can significantly influence fatigue performance.

**Figure 5:**
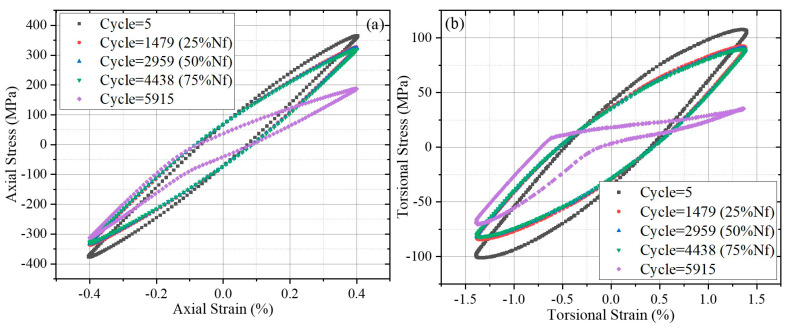
Hysteresis loops for AMPMF07 with different cycles: (**a**) axial hysteresis loops, (**b**) torsional hysteresis loops.

Figure 5 shows the axial and torsional hysteresis loops for AMPMF07 at different stages of the whole lifetime cycle. Axial and torsional hysteresis loops were selected from the 5th-cycle, 25%Nf-cycle, 50%Nf-cycle, 7%Nf-cycle, and 5915th-cycle. Significant softening occurred between the 5th-cycle and 1479th-cycle. The period from the 1479th-cycle to 4438th-cycle exhibited a stable cyclic stage. As for the 5915th-cycle, when final failure occurred, the hysteresis loop showed pronounced distortion. This distortion was primarily localized to the upper half of the loop, while the lower half remained relatively intact.

### 3.3. Mean Axial and Torsional Stress Response

**Figure 6:**
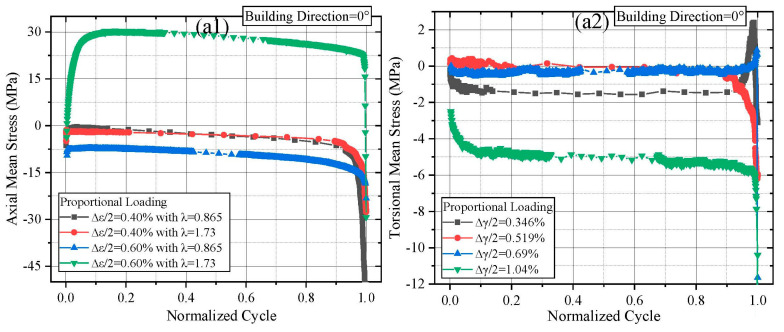

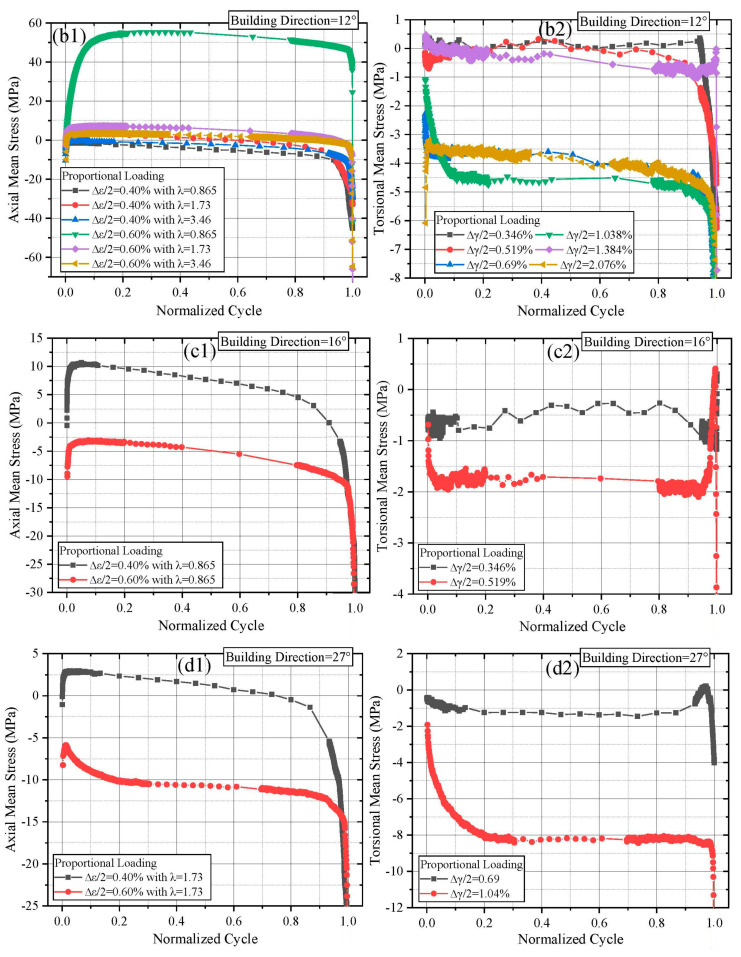
Axial and torsional mean stress response of L-PBF Ti64 with different building directions: (**a1**,**a2**) building direction = 0°, (**b1**,**b2**) building direction = 12°, (**c1**,**c2**) building direction = 16°, (**d1**,**d2**) building direction = 27°.

Figure 6 illustrates the axial and torsional mean stress response of L-PBF Ti64 with different building directions and different λ. As shown in Figure 6(a1,a2), only specimen AMPMF04 (0° building direction) exhibited a pronounced increase in axial mean stress. The mean stress rose rapidly during the initial cycles, stabilized, and then remained relatively constant until final failure. A similar trend was observed in specimen AMPMF08 (12° building direction). Considering the magnitude of the mean stress relative to the magnitude of the stress amplitude, none of the specimens built at 16° and 27° exhibited a significant axial mean stress response. Regarding torsional mean stress, specimens with noticeable mean stress development followed similar patterns as those observed in the axial channel—rapid initial accumulation followed by stabilization until failure.

From the perspective of multiaxial strain ratio, an increase in λ generally suppressed the axial mean stress while amplifying the torsional mean stress. A higher torsional component appeared to mitigate axial tensile–compressive asymmetry more rapidly. This asymmetric behavior is evident from the early rapid increase in mean stress followed by stabilization. This behavior may be attributed to the anisotropic microstructure inherent to L-PBF fabrication, which induces tension–compression asymmetry. However, unlike CM rolled titanium, which often shows continuous mean stress growth, L-PBF Ti64 specimens rapidly reached a steady state in mean stress. This suggests that the tensile–compressive asymmetry in L-PBF Ti64 is, to some extent, self-limiting and more controllable.

## 4. Macro- and Micro-Characterization and Fracture Behavior of L-PBF Ti64

Following the analysis of the mechanical behavior, this section focuses on the macro- and micro-scale deformation mechanisms of L-PBF Ti64 based on post-failure examinations.

### 4.1. Crack Propagation Angles of L-PBF Ti64

**Figure 7:**
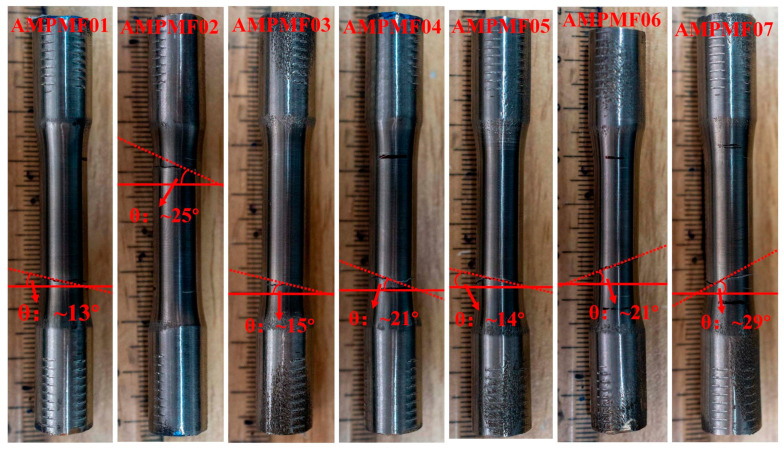

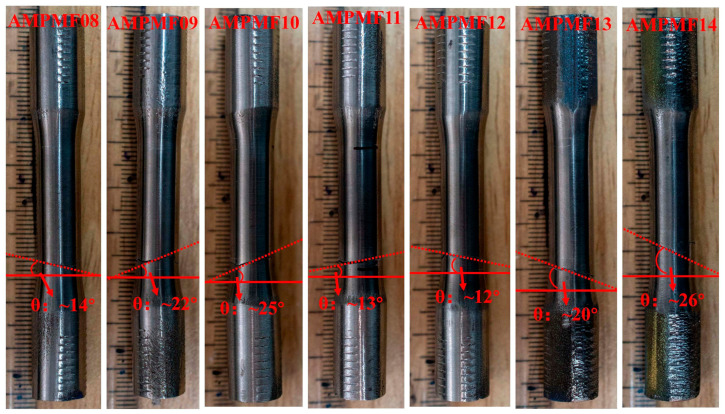
Crack propagation angle of L-PBF Ti64 with different building direction.

To determine the crack propagation angles after failure, high-resolution images of the crack projection surfaces were captured for each specimen using a 42.4-megapixel SONY Alpha 7RIII camera equipped with a 35 mm F1.8 lens, as presented in Figure 7.

The angle measurements in Figure 7 correspond to the identified crack propagation zone. The definition of these zones follows the same criteria used for the critical plane shown in the schematic in Figure 2. The measured results for each specimen are summarized in Table 5 and compared against both the experimentally observed fracture plane angles of rolled titanium and the theoretical critical plane angles calculated based on the maximum principal strain criterion.

These results demonstrate that the fracture plane angles in L-PBF Ti64, across all building directions, lie between the angles observed in rolled titanium and the theoretical critical plane angles predicted by the maximum principal strain criterion. This confirms the applicability of the critical plane theory to L-PBF Ti64 under multiaxial loading.

### 4.2. Fatigue Fracture Mechanism of L-PBF Ti64

**Figure 8:**
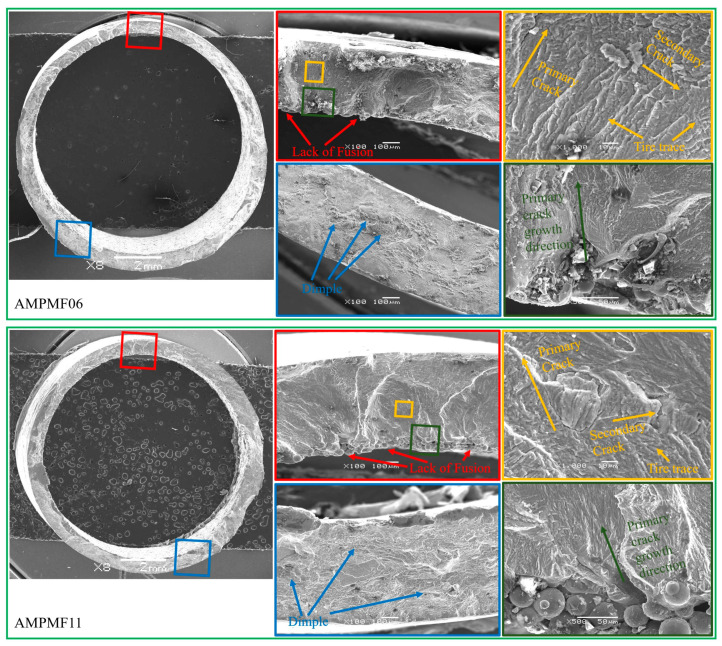

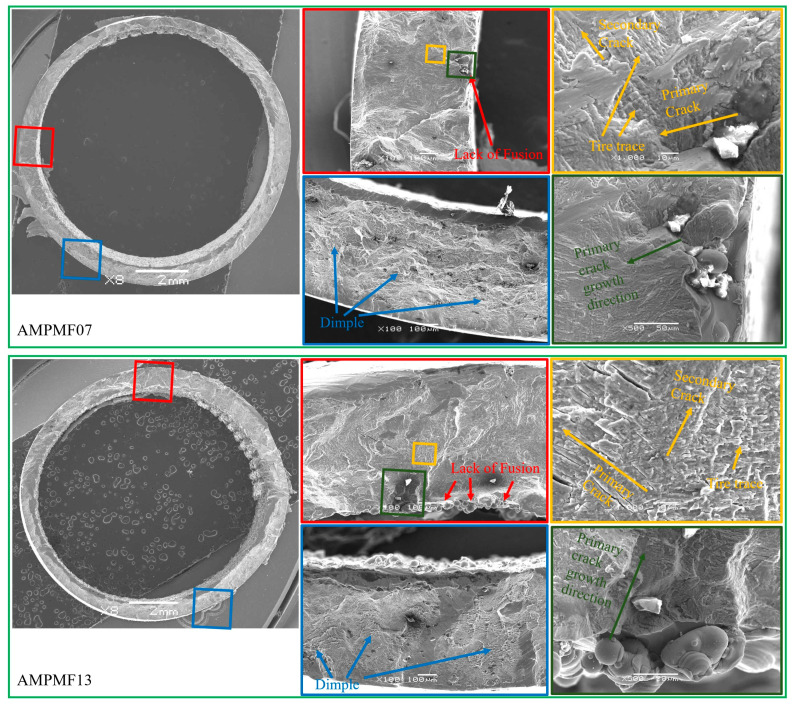
Fracture morphology of L-PBF Ti64.

To investigate the fatigue crack initiation and propagation behavior of L-PBF Ti64 under MLCF loading with varying multiaxial strain ratios and building directions, fracture surfaces of post-fatigue specimens were examined using a JEOL JSM-7800F field emission gun scanning electron microscope (SEM). Figure 8 presents the SEM images of the fatigue crack initiation zone, crack propagation zone, and final rupture zone for specimens tested at an axial strain amplitude of 0.4%, covering different multiaxial strain ratios and building directions.

Specimens AMPMF01 and AMPMF02 (0° building direction) exhibit a number of under-fused powder particles along the inner wall surface in the crack propagation zone. Fatigue cracks primarily initiate at the interface between lack-of-fusion (LOF) defects and the surrounding material, which act as inherent crack initiation sites. At 100× magnification, these LOF features are significantly more prevalent on the inner surface than the outer surface. Despite equal machining tolerances on both surfaces, the final surface quality of the inner wall remains inferior, making it more susceptible to crack initiation. Unlike the surface-driven crack propagation observed in rolled titanium, crack initiation from inner surfaces in L-PBF Ti64 represents a more critical failure mode, emphasizing the importance of investigating its multiaxial fatigue failure. At higher magnifications, typical fatigue features such as tearing ridges and river patterns are evident in both the initiation and propagation regions. Compared to AMPMF01, AMPMF02 presents a smoother surface in the crack propagation zone due to a higher multiaxial strain ratio. Significant fatigue striations in multiple directions can be observed in the crack propagation zone after magnification, as shown in the orange box. The primary crack growth direction along the radial direction and the secondary growth direction along the circumferential direction can be observed too. Furthermore, the tire trace formed by the superposition of fatigue striations generated in the two directions can be observed in the fracture morphology of L-PBF Ti64.

Specimens AMPMF05, AMPMF06, and AMPMF07 (12° building direction) demonstrate slightly improved inner-surface quality compared to those with 0° building direction. The overall fracture morphology characterization trend remained consistent, with the crack expansion zone consisting of tearing ridges and river patterns. The same tire trace can be observed in crack propagation zone. However, in the final rupture zone, more dimples can be observed in specimens under the 12° building direction compared to the 0° building direction. As the main microscopic feature of plastic fracture in metals, more dimples represent, to some extent, better plasticity.

Specimen AMPMF11 (16° building direction) exhibits fracture morphology highly comparable to AMPMF05. The pronounced cleavage and quasi-cleavage characteristics are closer to the fracture mechanism of rolled titanium after failure under similar applied multiaxial loading. A large number of dimples in the final rupture zone confirms the ductile nature of the failure. In contrast, specimen AMPMF13 (27° building direction) shows fracture features more akin to those of AMPMF02. The fatigue striation and tire trace in the crack propagation zone became finer and less distinct, suggesting a change in crack growth dynamics. Furthermore, the final rupture zone contains fewer dimples and more featureless regions, suggesting a subtle transition toward brittle fracture behavior, although most plastic characteristics are retained.

## 5. MLCF Life Prediction of L-PBF Ti64

Finally, MLCF life prediction models for L-PBF Ti64 with different building directions will be presented in this section.

### 5.1. Fatigue Life Distribution of L-PBF Ti64

**Figure 9:**
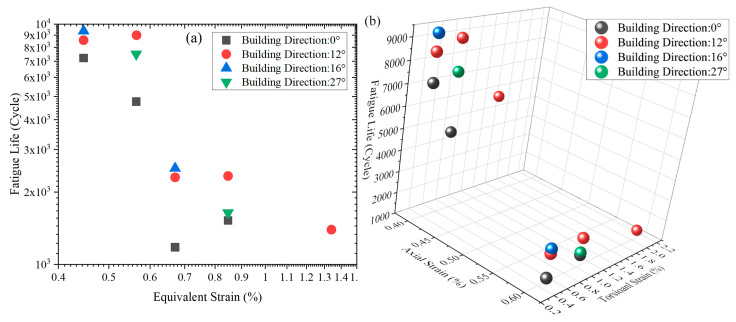
Fatigue life distribution of L-PBF Ti64 with different building directions: (**a**) equivalent strain—fatigue life, (**b**) axial strain—torsional strain—fatigue life.

Figure 9 presents both 2D and 3D distributions of the fatigue life of L-PBF Ti64 specimens. The 2D plots are based on von Mises equivalent strain, calculated under combined axial and torsional loading conditions using Equation (3) [34]:

(3) $$\varepsilon_{equiv}=\sqrt{{\left(\Delta \varepsilon /2\right)}^{2}+\frac{{\left(\Delta \gamma /2\right)}^{2}}{3}}$$

where Δε/2 and Δγ/2 are the axial and torsional strain amplitudes, respectively.

As shown in Figure 9a, under identical equivalent strain loading, specimens built at 0° exhibit the shortest fatigue lives. In contrast, those fabricated at 12° and 16° show significantly longer fatigue lives. For the two specimens with building direction of 27°, it was significantly higher under lower applied equivalent strain loading than 0°, and closer to the 0° under higher applied equivalent strain loading. These results suggest that aligning the building direction closer to the observed fracture plane inclination provides a more substantial life improvement than aligning with the theoretical maximum principal strain plane.

Considering that the equivalent strain blurs the boundary between axial and torsional applied strains, Figure 9b uses a 3D distribution to better visualize the interaction between axial and torsional loading components. In the 3D view, it can be seen that the MLCF life distribution of L-PBF Ti64 with different building directions is basically located in two large areas, the upper left area and the lower right area. The upper left corner represents the relatively high MLCF life region, where different torsional applied strain loadings have a significant effect on MLCF life of L-PBF Ti64. When coming to the low life region in the lower right corner, the effect of applied torsional strain loading on MLCF life is significantly reduced, and then the MLCF life of L-PBF Ti64 is basically controlled by the axial strain loading. This distributional characteristic shows the variation in the sensitivity of the MLCF life of L-PBF Ti64 to different levels of applied axial and torsional loadings.

### 5.2. MLCF Life Prediction Model

#### 5.2.1. Proposed KBMP Model with the Critical Plane Defined by the Maximum Principal Strain

**Figure 10:**
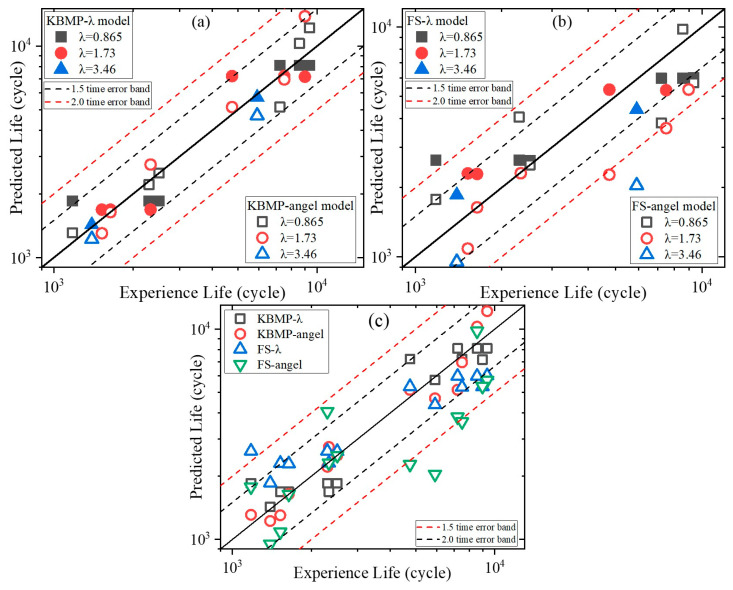
Comparisons between experimental life and predicted life predicted by the (**a**) KBMP-λ model and KBMP-angel model, (**b**) FS-λ model and FS-angel model, and (**c**) all models.

The original KBM model, developed by Brown and Miller [35], was founded based on the inference that multiaxial cracks are primarily controlled by the maximum shear strain and the normal strain on the plane of maximum shear strain. Kandil [36] later adapted this model for combined axial and torsional loading. Based on our past research [26,37], the original KBM model was modified to the proposed KBMP model to better accommodate the MLCF behavior of titanium, as shown in Equation (4):

(4) $$\frac{\Delta \gamma_{max}}{2}+S_{p}\Delta \varepsilon_{n}=\left[1+v_{e}+S_{p}\left(1-v_{e}\right)\right]\frac{\sigma_{f}^{\prime }}{E}{\left(2N_{f}\right)}^{b}+\left[1+v_{p}+S_{p}\left(1-v_{p}\right)\right]\varepsilon_{f}^{\prime }{\left(2N_{f}\right)}^{c}$$

where $\Delta \gamma_{max}$ and $\Delta \varepsilon_{n}$ are the maximum shear strain range and normal strain range on the critical plane, respectively. $v_{e}$ and $v_{p}$ are the elastic and plastic Poisson’s ratios, respectively. The values of $v_{e}$ and $v_{p}$ are 0.34 and 0.5, respectively [38]. The fatigue parameters $\sigma_{f}^{\prime }$, $\varepsilon_{f}^{\prime }$, b, and c in the KBMP model are aligned with uniaxial fatigue parameters of rolled CP-Ti to simplify model parameterization. $S_{p}$ is an influence coefficient of the normal strain in the critical plane defined by the maximum principal strain. Considering that the L-PBF Ti64 has an additional building direction variable, two approaches are taken for the fitting of $S_{p}$ in this paper: the multiaxial strain ratio-based fitting and the building direction-based fitting. All values of fatigue parameters are listed in Table 6. The fatigue lives predicted by the KBMP model based on these two fitting methods (KBMP-λ model and KBMP-angel model) are compared with experimental data in Figure 10a.

#### 5.2.2. Fatemi-Socie Model

To benchmark the performance of the prediction effectiveness of the KBMP model for L-PBF Ti64, the widely accepted FS model is used as a comparison model. Fatemi et al. [39] proposed this modified model based on the strain and stress parameters for shear failure mode material based on the following Equation (5):

(5) $$\frac{\Delta \gamma_{max}}{2}\left(1+k\frac{\sigma_{n}^{max}}{\sigma_{y}}\right)=\frac{\tau_{f}^{\prime }}{G}{\left(2N_{f}\right)}^{b_{0}}+\gamma_{f}^{\prime }{\left(2N_{f}\right)}^{c_{0}}$$

where $\Delta \gamma_{max}$, $\sigma_{n}^{max}$, and $\sigma_{y}$ are the maximum shear strain range and the maximum normal stress and yield stress in the maximum shear strain plane, respectively. k is a constant reflecting the effect of torsion. The right-hand side of Equation (5) may alternatively be expressed in terms of uniaxial fatigue properties as Equation (6) in the event that shear fatigue properties are not available for damage calculation [40].

(6) $$\frac{\Delta \gamma_{max}}{2}\left(1+k\frac{\sigma_{n}^{max}}{\sigma_{y}}\right)=\left[\left(1+v_{e}\right)\frac{\sigma_{f}^{\prime }}{E}{\left(2N_{f}\right)}^{b}+\left(1+v_{p}\right)\varepsilon_{f}^{\prime }{\left(2N_{f}\right)}^{c}\right]\left[1+k\frac{\sigma_{f}^{\prime }}{2\sigma_{y}}{\left(2N_{f}\right)}^{b}\right]$$

where $v_{e}$ is elastic Poisson’s ratio, $v_{p}$ is Poisson’s ratio for fully plastic conditions. Values of $v_{e}$ and $v_{p}$ are 0.34 and 0.5, respectively, and all other fatigue properties correspond to the fully reversed uniaxial strain-life equation. Similarly to the KBMP model, two approaches are taken for the fitting of the n-parameter. All values of fatigue parameters are listed in Table 6. The fatigue lives predicted by the FS model based on these two fitting methods (FS-λ model and FS-angel model) are compared with experimental data in Figure 10b.

### 5.3. Comparison of Life Prediction Performance of Different Models

As illustrated in Figure 10, both KBMP fitting strategies (λ-based and angle-based) outperform the corresponding FS variants. For the KBMP model, the prediction results basically lie within the 1.5 times error band. In contrast, the prediction results of the FS model are located in the 2 times error band, and there are even results beyond the 2 times error band. The full distribution of the four models can be visualized quite well in Figure 10c. The distributions of the FS models are further away from the center than the KBMP models.

**Figure 11:**
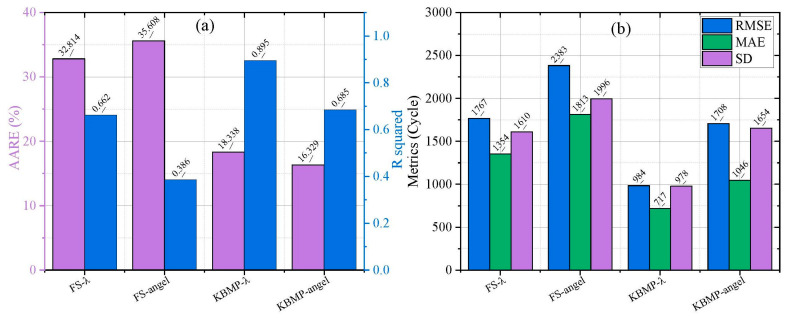
Quantitative error results of all models: (**a**) relative errors, (**b**) absolute errors.

In addition to the qualitative analysis of the life distribution predicted by each model, quantitative analysis is indispensable. R-Squared ($R^{2}$), average absolute relative error (AARE), root mean squared error (RMSE), mean absolute error (MAE), and standard deviation (SD) are used to indicate the predictive effectiveness of different models, which are given in Equations (7)–(11), as shown in Figure 11:

(7) $$R^{2}=\frac{\sum_{i=1}^{n}{\left(\log N_{pi}-\bar{\log N_{f}}\right)}^{2}}{\sum_{i=1}^{n}{\left(\log N_{fi}-\bar{\log N_{f}}\right)}^{2}}$$

(8) $$AARE=\frac{1}{n}\sum_{i=1}^{n}\left|\frac{\log_{10}N_{pi}-\log_{10}N_{fi}}{\log_{10}N_{fi}}\right|$$

(9) $$RMSE=\sqrt{\frac{1}{n}\sum_{i=1}^{n}{\left(\log N_{fi}-\log N_{pi}\right)}^{2}}$$

(10) $$MAE=\frac{1}{n}\sum_{i=1}^{n}\left|\log N_{fi}-\log N_{pi}\right|$$

(11) $$SD=\sqrt{\sum_{i=1}^{n}\frac{1}{n-1}{\left(\left|\frac{\log_{10}N_{pi}-\log_{10}N_{fi}}{\log_{10}N_{fi}}\right|-AARE\right)}^{2}}$$

Quantitative analysis confirms the superior performance of the proposed KBMP model over the FS model in predicting the MLCF life of L-PBF Ti64 across different building directions. Regarding the two fitting approaches for the KBMP model, the difference between the KBMP-λ model and KBMP-angel model is mainly reflected in the fact that the AARE of the KBMP-angel model is better while the other four error indicators of the KBMP-λ model are better. The KBMP-angel model does not perform as well as the KBMP-λ model in terms of absolute error, but the relative error is the lowest of all models, suggesting that it predicts small-valued samples more accurately. Overall, the KBMP-λ model is recommended for general MLCF life prediction, especially in higher-life regimes, due to its comprehensive optimality, while the KBMP-angel model is more accurate for lower-life predictions.

## 6. Conclusions

(1) L-PBF Ti64 exhibits characteristic three-stage cyclic softening behavior (continuous softening, stabilization, and rapid failure). Both the building direction and the multiaxial strain ratio (λ) significantly influence the stress response, with the 12° building direction demonstrating the highest stress amplitude and thus superior fatigue resistance.
(2) The fracture surface inclinations of L-PBF Ti64 specimens across all building directions lie between the angles observed in conventionally rolled titanium and the theoretical values predicted by the critical plane theory based on the maximum principal strain criterion. Critical plane theory remains applicable to L-PBF Ti64 under multiaxial loading.
(3) Fatigue cracks of L-PBF Ti64 predominantly initiate from inner-surface LOF defects. Crack propagation shows tearing ridges, river patterns, and multi-directional striations, with radial primary growth and circumferential secondary growth. The 12° specimens exhibit improved inner-surface quality and a higher prevalence of ductile dimples compared to the 0° specimens. For L-PBF Ti64 under MLCF, aligning the building direction with the experimentally observed fracture plane, rather than the theoretical maximum principal strain plane, leads to enhanced fatigue performance.
(4) The proposed KBMP-λ model demonstrates superior predictive capability for MLCF life across different building directions compared to the traditional FS model. As a pure strain parameter-based model, it requires only loading parameters as input while effectively accounting for the building direction effect, enabling high prediction accuracy under strain-controlled conditions. The KBMP-λ model achieves excellent performance in absolute error metrics, making it particularly suitable for middle-cycle life predictions in engineering applications. In contrast, the KBMP-angel model provides higher accuracy for low-cycle life predictions, ensuring robust performance across the entire fatigue life spectrum.

## Figures and Tables

**Table 1:** Process parameters of infill and contour regions provided by EOS.

	Laser Power [W]	Scanning Speed [mm/s]	Hatching Distance [μm]	Layer Thickness [μm]
Infill	280	1200	140	30
Contour	150	1250	/	30

**Table 2:** Tensile test data of L-PBF Ti64.

Specimen No.	Building Direction [°]	$\mathrm{Tensile}\;\mathrm{Strength}/\sigma_{u}$ [MPa]	$\mathrm{Yield}\;\mathrm{Strength}/\sigma_{y}$ [MPa]	$\sigma_{u}/\sigma_{y}$	Elastic Modulus [GPa]
T0-01	0	1286.26	1104.15	1.165	102.42
T0-02	0	1247.25	1100.21	1.134	105.43
T12-01	12	1223.92	1021.55	1.198	106.50
T12-02	12	1290.64	1103.26	1.170	109.51
T16-01	16	1238.45	1054.59	1.174	107.29
T16-02	16	1106.65	926.66	1.194	108.77
T27-01	27	1225.51	1055.88	1.161	102.40
T27-02	27	1169.17	1008.81	1.159	102.35

**Table 3:** LCF test data of L-PBF Ti64.

Specimen No.	Building Direction [°]	Axial Strain Amplitude [%]	Axial Stress Amplitude [MPa]	Nf [Cycle]
AMAF01	0	0.40	421.18	11,421
AMAF02	0	0.6	649.08	2801
AMAF03	0	0.8	879.83	2069
AMAF04	12	0.4	457.75	8794
AMAF05	12	0.6	650.36	3933
AMAF06	12	0.8	744.02	1546
AMAF07	27	0.4	437.07	9975
AMAF08	27	0.6	639.49	4062
AMAF09	27	0.8	812.73	2140

**Table 4:** MLCF test data of L-PBF Ti64.

Specimen No.	Building Direction [°]	Strain Amplitude [%]		Stress Amplitude [MPa]		λ	Nf [Cycle]
		Axial	Torsional	Axial	Torsional		
AMPMF01	0	0.4	0.346	333.85	22.02	0.865	7217
AMPMF02	0	0.4	0.690	296.60	38.31	1.73	4752
AMPMF03	0	0.6	0.519	474.08	31.23	0.865	1178
AMPMF04	0	0.6	1.038	479.66	63.77	1.73	1523
AMPMF05	12	0.4	0.346	341.52	21.56	0.865	8562
AMPMF06	12	0.4	0.692	338.23	43.81	1.73	8982
AMPMF07	12	0.4	1.384	328.69	86.52	3.46	5918
AMPMF08	12	0.6	0.519	476.17	30.88	0.865	2301
AMPMF09	12	0.6	1.038	502.79	67.82	1.73	2332
AMPMF10	12	0.6	2.076	456.82	123.28	3.46	1394
AMPMF11	16	0.4	0.346	327.93	42.67	0.865	9352
AMPMF12	16	0.6	0.519	436.46	29.14	0.865	2510
AMPMF13	27	0.4	0.690	264.34	17.23	1.73	7503
AMPMF14	27	0.6	1.038	390.44	53.50	1.73	1641
AMPMF02-R	0	0.4	0.690	-	-	1.73	5003
AMPMF06-R	12	0.4	0.692	-	-	1.73	8164
AMPMF10-R	12	0.6	2.076	-	-	3.46	1176

**Table 5:** Crack propagation and critical plane angle of L-PBF Ti64.

Specimen No.	Building Direction [°]	Fracture Surface Inclination of Rolling Ti [°]	Critical Plane Based on the Maximum Principal Strain [°]	Fracture Surface Inclination of L-PBF Ti64 [°]
AMPMF01	0	12.0	16.9	~13
AMPMF02	0	17.9	26.6	~25
AMPMF03	0	12.0	16.9	~15
AMPMF04	0	17.9	26.6	~21
AMPMF05	12	12.0	16.9	~14
AMPMF06	12	17.9	26.6	~21
AMPMF07	12	23.5	34.8	~29
AMPMF08	12	12.0	16.9	~14
AMPMF09	12	17.9	26.6	~22
AMPMF10	12	23.5	34.8	~25
AMPMF11	16	12.0	16.9	~13
AMPMF12	16	12.0	16.9	~12
AMPMF13	27	17.9	26.6	~20
AMPMF14	27	17.9	26.6	~26

**Table 6:** Values of various fatigue parameters for different models.

λ	$\mathrm{KBMP}/S_{p}$	FS/k	Building Direction [°]	$\mathrm{KBMP}/S_{p}$	FS/k
0.865	1.30	−0.057	0°	1.44	−0.051
1.73	1.36	−0.065	12°	1.24	−0.065
3.46	1.21	−0.084	16°	1.20	−0.056
			27°	1.37	−0.058

## Data Availability

The original contributions presented in this study are included in the article. Further inquiries can be directed to the corresponding author.

## Abbreviations

The following abbreviations are used in this manuscript:

AARE	Average absolute relative error
AM	Additive manufacturing
CM	Conventional manufacturing
LCF	Low-cycle fatigue
LOF	Lack of fusion
L-PBF	Laser powder bed fusion
MAE	Mean absolute error
MLCF	Multiaxial low-cycle fatigue
RMSE	Root mean squared error
SD	Standard deviation
SEM	Scanning electron microscopy
Ti64	Ti-6Al-4V
